# A Combined Approach for Detection of Ovine Small Ruminant Retrovirus Co-Infections

**DOI:** 10.3390/v15020376

**Published:** 2023-01-28

**Authors:** Giuliana Rosato, Carlos Abril, Monika Hilbe, Frauke Seehusen

**Affiliations:** 1 Institute of Veterinary Pathology, Vetsuisse Faculty, University of Zurich, CH 8057 Zurich, Switzerland; 2 Institute of Virology and Immunology IVI, in Cooperation with the Vetsuisse-Faculty of the University of Bern, 3012 Bern, Switzerland

**Keywords:** MVV, CAEV, JSRV, ovine pulmonary adenocarcinoma (OPA), diagnostic, co-infections, viral cell tropism

## Abstract

Jaagsiekte retrovirus (JSRV)-induced ovine pulmonary adenocarcinoma (OPA) is an important ovine respiratory disease in Switzerland. Furthermore, ovine lungs with OPA frequently exhibited lesions suggestive of maedi-visna virus (MVV) or caprine arthritis encephalitis virus (CAEV) infection, indicating that co-morbidities might occur. Lungs and pulmonary lymph nodes were sampled from suspected OPA cases, inflammatory lung lesions and control lungs (total of 110 cases). Tissues were (a) processed for histology and immunohistochemistry (IHC), and (b) underwent DNA extraction and real-time PCR for JSRV, MVV and CAEV. Peptide sequences were used to generate virus-specific customized polyclonal antibodies. PCR-positive OPA cases and formalin-fixed and paraffin-embedded MVV- and CAEV-infected synovial cell pellets served as positive controls. Fifty-two lungs were histologically diagnosed with OPA. Histological evidence of MVV/CAEV infection was detected in 25 lungs. JSRV was detected by PCR in 84% of the suspected OPA cases; six were co-infected with MVV and one with CAEV. MVV was detected by PCR in 14 cases, and four lungs were positive for CAEV. Three lungs had MVV/CAEV co-infection. In IHC, JSRV was detected in 91% of the PCR-positive cases, whereas MVV and CAEV immunoreactivity was seen in all PCR-positive lungs. Although PCR showed a higher sensitivity compared to IHC, the combined approach allows for investigations on viral cell tropism and pathogenic processes in co-morbidities, including their potential interdependency. Furthermore, an immunohistochemical tool for specific differentiation of MVV and/or CAEV infection was implemented.

## 1. Introduction

Retroviruses are enveloped, single-stranded, positive-sense RNA viruses (80–100 nm in diameter) that cause various diseases, including neoplasia and immunological conditions. The *Retroviridae* family is subdivided into two subfamilies, the *Orthoretrovirinae* and the *Spumaretrovirinae*. Enclosed in the *Orthoretrovirinae* subfamily, seven genera exist: Alpharetrovirus, Betaretrovirus, Deltaretrovirus, Epsilonretrovirus, Gammaretrovirus and Lentiviruses. Within this subfamily, small ruminant lentiviruses (SRLVs), the Betaretroviruses Jaagsiekte sheep retrovirus (JSRV) and enzootic nasal tumor virus (ENTV) infect small ruminants and cause diseases, such as gradually progressing inflammations for SRLV and neoplastic diseases for JSRV and ENTV, respectively, leading to important economic and welfare implications in sheep-rearing countries [1,2].

The SRLV group comprise maedi-visna virus (MVV) and caprine arthritis encephalitis virus (CAEV), originally isolated in sheep and goats, respectively. MVV and CAEV were described to be two different strictly host-specific viruses, depending on the infected species (sheep versus goats). Phylogenetic studies about partial sequences of SRLV have revealed that cross-species transmission occurs [3,4,5] and there is also demonstration of co-infection of single animals, resulting in recombination among viral variants [6]. SRLVs show major tropism for monocyte/macrophages and dendritic cells [7,8,9,10,11]. Nevertheless, other cell types, including mammary epithelial cells, may well be infected and act, therefore, as reservoirs of the virus. These cells in the mammary gland are a significant source of infected cells and free virus for vertical transmission of SRLVs from dams to their offspring by ingestion or seldom aspiration of infectious colostrum [12,13]. The horizontal route of infection is erogenous (direct contact or expiration of infectious secretions) to the respiratory tract or conjunctiva of naïve animals [14,15,16,17]. Horizontal infection between animals through contaminated milking machines is also described [18]. The four principal tissues affected by SRLV are the lungs, mammary gland, CNS and joints. MVV infection in sheep leads to lympho-plasmacytic interstitial pneumonia, formation of lymphoid nodules with germinal centers, hypertrophy of smooth muscles in the alveolar septa and interstitial fibrosis. In the affected CNS, meningoencephalitis, astrocytosis, microgliosis and demyelination occur in both the brain and spinal cord. Additionally, SRLV-infected small ruminants show a non-suppurative mastitis and chronic arthritis characterized by a mononuclear cell infiltration and villous hypertrophy of the synovial membrane of the carpal or tarsal joints [2,19,20,21].

JSRV is an oncogenic virus that causes neoplastic transformation of lung epithelial cells leading to OPA; the virus is found worldwide and reported in numerous countries across America, Asia, Africa and Europe.

Natural transmission of JSRV occurs primarily via the erogenous route through inhalation of cell-free particles, but transuterine [22] or oral transmission via infected colostrum and milk probably also play a role. Several studies showed that JSRV infection can occur perinatally or in the first life months in lambs as well as in adult sheep [23,24,25]. JSRV can infect lung epithelial cells as well as lymphocytes and cells of the monocyte/macrophage system [26].

The incubation period of OPA in sheep is up to years and symptoms are mostly reported in adults aged 2–4 years. Clinical symptoms consist of coughing, nasal discharge of clear frothy fluid that can be induced with the colloquially referred “wheelbarrow-test” and loss of condition with exercise intolerance [25,27]. On post mortem examination, detection of characteristic macroscopic and histologic lesions of OPA is the standard method for the diagnosis of JSRV infection in single animals or in a flock [27]. Polymerase chain reaction (PCR) and immunohistochemistry (IHC) are useful methods to detect the viral nucleic acid and proteins in animals with early-stage lesions that have not yet fully developed OPA. Additionally, these methods can be used as follow-up tests to confirm an infection in unclear cases. Typical lesions consist of neoplastic transformation and proliferation of type II pneumocytes and bronchial epithelial cells, with rare metastasis to the regional lymph nodes and further organs [28].

Results of a project on gross changes in organs of slaughtered animals alerted us of the likely underreporting of JSRV-induced OPA in Switzerland [1]. Between 2012 and 2016, before the project was started, 2 to 12 cases per year were notified to the Federal Food Safety and Veterinary Office. In contrast, during implementation of the project in 2019, 56 cases were reported to the authorities in that particular year (Supplementary Figure S1). Furthermore, ovine lungs with OPA frequently exhibited histologic lesions suggestive of MVV or CAEV infection, indicating that co-morbidities might occur [2,29,30]. In Switzerland, the reported prevalence of both notifiable diseases OPA and MV/CAE is quite low.

The ongoing SRLV diagnostic strategy in Switzerland is based on an array of four sequentially used serological tests [31] on single animals. Recently, a nested real-time PCR protocol with the ability to differentiate between CAEV and MVV was developed [32]. Confirmation of the disease in basic necropsy protocols in the pathology field requires the detection of the causative viruses, optimally both at molecular and in situ level. To our knowledge, commercial antibodies that can differentiate between MVV and CAEV are not yet available.

This study was performed to (i) optimize the diagnostic tools for the detection of notifiable ovine pulmonary diseases caused by JSRV, MVV and CAEV such as PCR but mainly to design antibodies for IHC, which are not commercially available; (ii) investigate viral cell tropism and pathogenic processes, including co-infections and their potential interference in lesion development; (iii) gain data on prevalence of ovine pulmonary viral diseases and their co-infection in Switzerland.

## 2. Materials and Methods

### 2.1. Animals and Tissue Samples

The study was performed on 110 lung tissue samples and—if available—pulmonary lymph nodes of Swiss sheep suspected for OPA and/or showing chronic pulmonary inflammatory lesions on macroscopical examination. The selected animals underwent a diagnostic post mortem examination after they had been euthanized (*n* = 35) due to clinical disease or were slaughtered (*n* = 29) between January 2018 and April 2022. Additionally, 33 lungs were collected from slaughtered sheep by the slaughterhouse in Zurich, Switzerland, and examined at the Institute of Veterinary Pathology, Vetsuisse Faculty, University of Zurich. Archival disease-free pulmonary samples (*n* = 13) were included as negative controls. The lungs were examined at the Institute of Veterinary Pathology, Vetsuisse Faculty, University of Zurich. For histological examination, pulmonary tissue samples of the slaughtered animals were collected systematically from all lung lobes and pulmonary lymph nodes. Samples were fixed in 10% buffered formalin for 24–48 h at room temperature for further histological and immunohistochemical examination or were immediately frozen in liquid nitrogen and stored at −80 °C for subsequent molecular examination. Sixty-four of the cases were archival diseased pulmonary samples.

### 2.2. Tissue Processing and DNA Extraction

DNA extraction of 30 mg of frozen and formalin-fixed paraffin-embedded tissue samples was performed using the DNeasy^®^ Blood & Tissue kit and the QIAamp DNA FFPE Tissue kit, respectively. Finally, DNA was eluted in 100 µL of elution buffer following the manufacturer’s instructions.

### 2.3. Nested Real-Time PCR for the Proviral Detection of the Exogenous JSRV (exJSRV), CAEV and MVV

**Figure 1:**
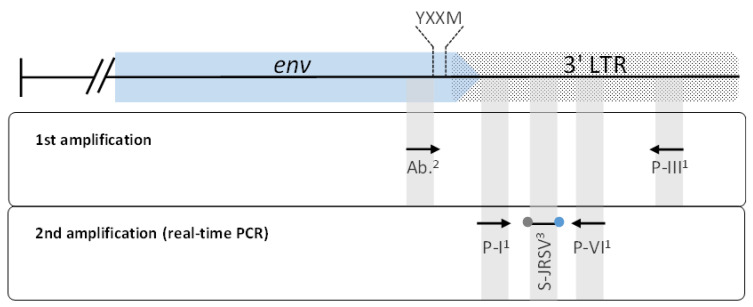
Schematic diagram showing the target regions of primers and probe of the exJSRV real-time PCR. These target sequences are located at the envelope protein gene (env) (blue arrow) and the 3′ long terminal repeat (LTR) (dotted box). ^1^ Primers described by Palmarini et al. [33], ^2^ primer designed by Abril et al. (manuscript in preparation), ^3^ fluorescent probe described by Bahari et al. [36]. The localization of the coding sequence for the YXXM motif is shown with dashed lines.

The exJSRV-PCR primers used in this work were originally designed based on Palmarini et al. [33] for the detection of the exogenous Jaagsiekte sheep retrovirus (exJSRV) provirus in infected animals. Briefly, the first round of PCR was performed with a newly designed forward primer targeting 12 base pairs immediately upstream of the coding sequence of the YXXM motif described by Liu and Miller [34] and the modified reverse primer PIII described by Palmarini et al. [33]. These 12 base pair sequences and the YXXM motif are exclusively found in the exJSRV [35]. Subsequently, a nested real-time PCR using modified primers PI and PVI described by Palmarini et al. [33] and the probe described by Bahari et al. [36] was performed (Figure 1). The amplification steps for the exJSRV-PCR nested real-time PCR and the nested real-time PCRs for CAEV and MVV were carried out as described by Schaer et al. [32]. The exJSRV real-time PCR for paraffin-embedded tissue samples was validated with tissue samples of 40 histopathological confirmed cases of OPA.

### 2.4. Histopathology

For histopathology, collected tissue samples were formalin fixed for 24 to 48 h and then routinely processed for paraffin embedding. Afterwards, 2 μm sections were stained with hematoxylin and eosin (HE) according to standard procedures.

### 2.5. Immunohistochemistry (IHC)

After being tested for viral DNA by nested real-time PCR, selected cases were further examined by immunohistochemistry with a panel of primary antibodies specific for several antigens to phenotype different cell populations, including pan-cytokeratin (PCK 26; marker for endothelial cells), synaptophysin (marker for neuroendocrine cells), Iba1 (marker for macrophages), surfactant protein C (SF-C; marker for type II pneumocytes), CD3 and CD 20 (marker for T and B lymphocytes, respectively) (Table 1).

For double immunostaining, detection of viral antigens was combined with cellular antigens (PCK 26, Iba1 and SP-C). Immunostaining for both antigens was performed sequentially. Visualization of the antigens was carried out by using either HistoGreen (Biozol, LIN-E109) or AEC (Zytomed Systems, ZUC037) as a substrate.

### 2.6. Protein Sequence Analysis and Production of Polyclonal Antibody against the Envelope Proteins of exJSRV, CAEV and MVV

Pairwise protein sequence alignments for the identification of conserved regions in the envelope (Env) proteins of exJSRV, the MVV and CAEV (sequences retrieved from the National Centre for Biotechnology Information, NCBI) were conducted using the Geneious prime software version 2020 (https://www.geneious.com, accessed on 6 December 2021). The region of interest to produce polyclonal antibodies against exJSRV was chosen according to Liu et al. [37]. The prediction of antigenic epitopes was conducted using the Antibody Epitope Prediction tools of the Immune Epitope Database and Analysis Resource (http://www.iedb.org/home_v3.php) and the Antigen Profiler tool from ThermoFisher Scientific (https://www.thermofisher.com/ch/en/home/life-science/antibodies/custom-antibodies/custom-antibody-production/antigen-profiler-antigen-preparation.html, accessed on 20 January 2021) (Supplementary Figure S2). Epitope cluster analysis (http://www.iedb.org/home_v3.php) was used to assess and visualize the conservancy of the epitope regions (Supplementary Figure S2). The suitability of the selected peptide sequences for production and purification was analyzed using the peptide synthesis and proteotypic peptide analyzing tool from ThermoFisher Scientific (https://www.thermofisher.com/us/en/home/life-science/protein-biology/peptides-proteins/custom-peptide-synthesis-services/peptide-analyzing-tool.html, accessed on 20 January 2021) (not shown). The selection of peptides was based on the conducted in silico assessments for antigenicity, conservancy and suitability for the synthetic production. Finally, the selected peptides corresponded to the amino acid residues exJSRV-Env596-614, CAEV-Env860-879 and MVV-Env860-876 based on the GenBank accession numbers: AFM29008.1, NP_040942 and ALU34108.1, respectively. Customized rabbit Polyclonal Antibodies were obtained from ThermoFisher Scientific (Table 1). Immunohistochemical investigation for the three viruses was performed on all 110 lungs. PCR-positive OPA cases with typical pathomorphological lesions as well as goat synovial membrane cells (P3) infected with either MVV or CAEV served as positive controls.

### 2.7. Macroscopic Examination

Of the 33 cases collected by the slaughterhouse in Zurich, gross lesions present on each lung lobe were recorded and classified based on common gross pathological changes, including abscesses, cranioventral consolidation, fibrosis, firm nodules, mineralization, parasitic granuloma, and macroscopic suspects of OPA. The macroscopic lesions from the diagnostic cases were recorded from the archival diagnostic necropsy reports and classified as described above.

### 2.8. Histologic Categorization of Lesions

All 97 altered lungs were categorized according to the histological lesion pattern present, as previously described in the literature [21,38]. A diagnosis of JSRV infection was made by the presence of OPA lesions consisting of typical neoplastic changes (single or multifocal, well-demarcated and infiltrative tumors with papillary/lepidic/acinar growth patterns consisting of cuboidal to columnar cells on a fine fibrous stroma and surrounded by histiocytic infiltration). The cases showing presence of chronic inflammatory lung lesions consistent with SRLV-like lesions (lymphohystiocytic and plasmacytic interstitial infiltrates, formation of lymphoid nodules with germinal centers, hypertrophy of smooth muscle in the alveolar septa, interstitial fibrosis and intraalveolar densely eosinophilic fluid) were defined as suspicious for SRLV/MVV/CAEV infection.

### 2.9. Statistical Analysis

Data were analyzed using IBM SPSS Statistics software version 28 (IBM, Armonk, NY, USA). Non-parametric analyses were performed using the Pearson’s chi square test to examine the association of/between the following parameters: PCR-positive lungs compared to IHC-positive lungs for JSRV, MVV and CAEV, respectively.

## 3. Results

### 3.1. Macroscopic Examination

**Figure 2:**
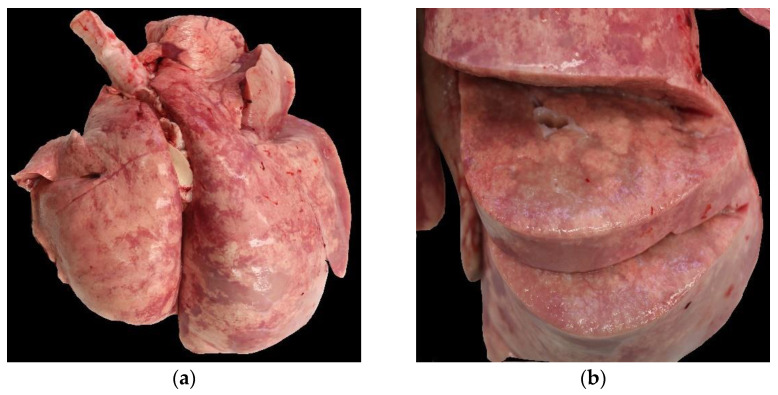
Macroscopical findings in a case with OPA. (**a**) Asymmetry and enlargement of the right caudal lobe. (**b**) Cut section with multiple, dense, greyish nodules.

On gross examination, 91 of the 97 lungs (without control group) showed single or multiple macroscopic lesions. Thus, 52 lungs were classed as “macroscopically suspected OPA”, which represented 53% of the examined lungs; thereof, 17 lungs were associated with other gross lesions. An example of a typical OPA lesion lung is depicted in Figure 2. No macroscopic lesions were reported in six archival cases. The second-most-detected macroscopic lesion was cranioventral consolidation, which was present in 33 lungs. Single or multiple pulmonary abscesses were seen in 12 cases. Other macroscopic findings, such as subpleural parasitic granulomas, pleural fibrosis and focal firm nodule formation suspicious of calcification, were diagnosed in single cases, mostly in the context of other lesions.

### 3.2. Histologic Examination

All 52 lungs macroscopically classified as suspected OPA histologically showed lesions of neoplasia and were, therefore, diagnosed as OPA.

Histological evidence of SRLV (MVV/CAEV) infection was seen in 25/97 cases.

**Figure 3:**
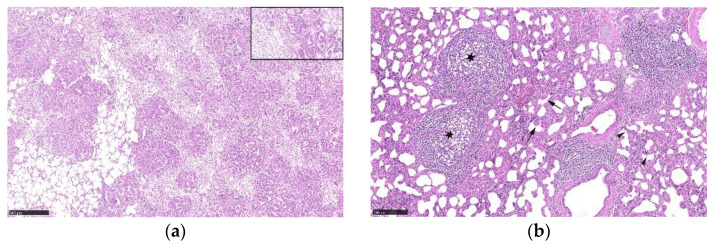
(**a**) Histological lesions of OPA. Infiltrative adenocarcinoma with acinar growth pattern on a fibrous stroma. Insert (top right): numerous intraalveolar macrophages are present around neoplastic epithelial cells. (**b**) Histological lesions compatible with an SRLV/MVV/CAEV infection. Lymphohistiocytic and plasmacytic interstitial infiltrates, formation of lymphoid nodules (asterisk), hypertrophy of smooth muscle in the alveolar septa (arrow) and interstitial fibrosis (arrowhead).

Histological lesions of an OPA-positive lung tissue are illustrated in Figure 3a. The 13 macroscopically normal control lungs were also investigated histologically and showed no significant pulmonary lesions. An example of lung tissue with histologic lesions compatible with a SRLV(MVV/CAEV) infection is illustrated in Figure 3b.

### 3.3. Nested Real-Time PCR for JSRV and SRLV (MVV/CAEV)

Of the 52 cases with gross and histologic lesions consistent with OPA, JSRV was detected by PCR in 44 cases (84%; 45% of all cases). Of these, six were co-infected with MVV and one with CAEV, respectively.

Of the 25 cases with histological lesions compatible with an SRLV infection, MVV was detected by PCR in six cases (24%), of which four were co-infected with JSRV.

MVV/CAEV were detected by PCR in 10 cases that were classified as having no histological lesions consistent with an SRLV infection. Of these cases, MVV was detected by PCR in nine cases, of which four were co-infected with CAEV and three co-infected with JSRV. None of the cases revealed a single infection with CAEV. Of the three MVV/CAEV co-infected lungs, two showed no macroscopic lesions and one had gross and histological evidence of parasitic granulomas. Thus, 45 lungs tested negative for all three viruses (Table 2).

### 3.4. Immunohistochemical Investigations

#### 3.4.1. JSRV

**Figure 4:**
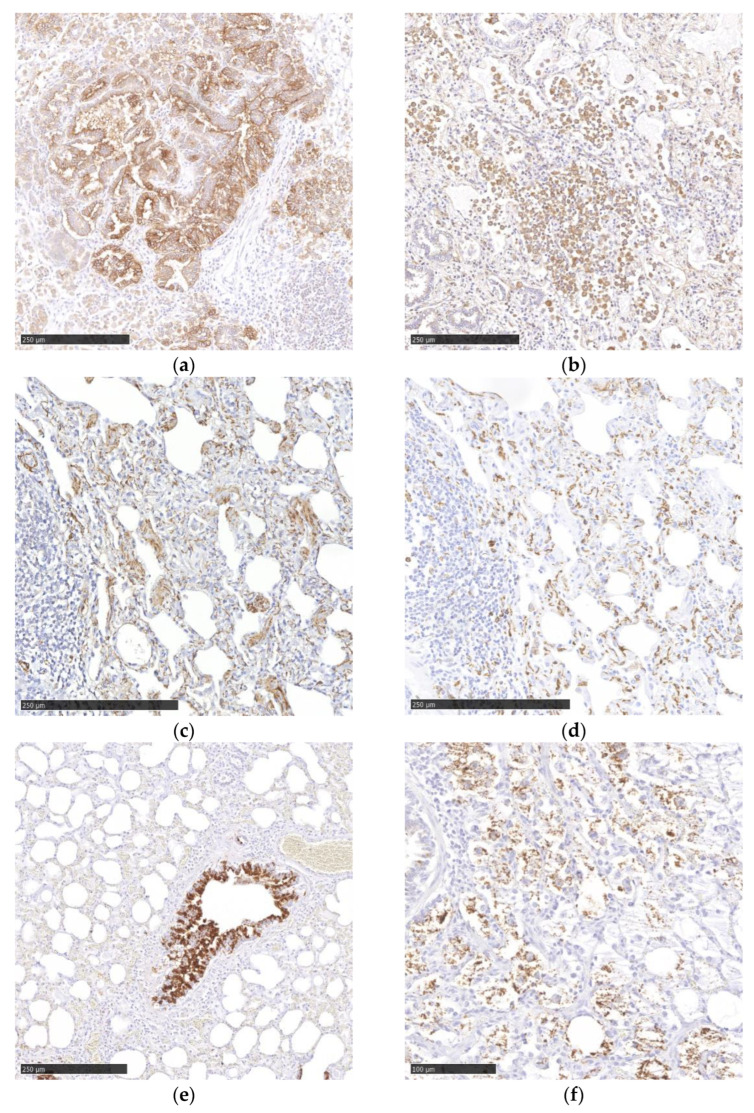
Immunohistochemistry to identify viral antigen and cell populations (**a**) JSRV antigen in epithelial cells. (**b**) JSRV antigen in alveolar macrophages. (**c**) MVV antigen in interstitial cells (**d**) Iba1 antigen in an MVV-positive lung. (**e**) CAEV antigen in bronchial epithelial cells. (**f**) CAEV antigen in macrophages. Chromogen DAB.

JSRV was detected by IHC in 91% (40/44) of the PCR-positive cases. The examination revealed virus antigen in the cytoplasm of neoplastic bronchial epithelial cells (Figure 4a) and in alveolar macrophages, demonstrated by immunohistochemical labeling for Iba1 (Figure 4b). In consecutive sections, many JSRV-infected cells were also PCK 26 positive and, therefore, interpreted as bronchial epithelial cells. Additionally, two animals with OPA showed PCK 26-positive neoplastic cells (interpreted as metastasis) in the pulmonary lymph node. In consecutive sections, only a small number of JSRV-infected cells showed immunoreactivity for SF-C (marker for type II pneumocytes). Synaptophysin IHC did not reveal any viral infection of neuroendocrine cells (Table 3).

#### 3.4.2. SRLV

In a first round of this investigation, only one localization of pulmonary tissue of each case was used. This resulted in 79% and 25% of IHC-positive cases in MVV and CAEV PCR-positive cases, respectively. After further examination of two additional localizations, MVV and CAEV, immunoreactivity was seen in 100% (14/14 and 4/4) of PCR-positive lungs, respectively.

In MVV IHC, cytoplasmic viral antigen was detected in hypertrophic type II pneumocytes (Figure 4c). Immunohistochemical labeling of MVV-positive lungs for Iba1 revealed the presence of cytoplasmic viral antigen in a moderate number of interstitial macrophages (Figure 4d). Immunohistochemical labeling of the same lung tissue for SF-C and PCK 26 revealed the presence of virus in a small number of type II pneumocytes and epithelial cells, respectively (Table 3).

In CAEV IHC, scattered bronchial epithelial cells (Figure 4e), a small number of macrophages (Figure 4f) within the alveoli and few type II pneumocytes showed the presence of cytoplasmic viral antigen (Table 3).

Immunohistochemical labeling of selected MVV and CAEV cases for CD3 and CD20 revealed the presence of a high amount of both T and B lymphocytes in lymphoid nodules and in the interstitial inflammatory infiltrates.

In the double IHC, a colocalization of the JSRV and Iba1 antigen in a moderate number of cells with a histiocytic morphology could be detected. Additionally, single cells were double positive for JSRV and SP-C (Supplementary Figure S3).

Furthermore, in MVV-infected lungs, a colocalization of MVV antigen and the macrophage marker Iba1 in a moderate number of cells was detected (Supplementary Figure S3).

The majority of PCR-negative lungs also did not display immunoreactivity in the IHC (Supplementary Figure S4).

### 3.5. Statistical Analysis

Pearson’s chi square test showed a significant association between JSRV PCR-positive lungs and JSRV IHC-positive lungs (minimum expected frequency 18.14; Pearson′s chi square value 81.994, *p* < 0.001). This association was also present between MVV PCR-positive lungs and MVV IHC-positive lungs (minimum expected frequency 2.02; Pearson’s chi square value 97.000, *p* < 0.001) as well as CAEV PCR-positive lungs and CAEV IHC-positive lungs (minimum expected frequency 0.16; Pearson’s chi square value 97.000, *p* < 0.001), respectively.

## 4. Discussion

In this study, ovine lungs from suspected OPA cases and inflammatory lung lesions were examined for infection with SRLV and JSRV. The aim was to optimize diagnostic tools such as PCR for the detection of notifiable ovine pulmonary diseases caused by JSRV, MVV and CAEV, and to design antibodies for IHC to investigate the viral cell tropism and pathogenic processes, including co-infections and their potential interference in lesion development and to gain data on the prevalence of ovine pulmonary viral diseases and their co-infection in Switzerland.

Retroviral diseases in the Swiss sheep population are notifiable diseases. Official data from the last few years revealed a low prevalence rate, except for a short period of time when the Federal Food Safety and Veterinary Office (FSVO) financially supported a project on changes in organs of slaughtered animals (from mid-2017 to end of 2019), which led to a rapid increase in OPA cases and histological suspicion of SRLV infection in ovine lungs. Thus, in these two years, 53% of all cases from the last 10 years were diagnosed. These findings reflect the necessity of a thorough investigation to increase the number of reports of notifiable diseases. In our opinion, OPA and SRLV infections appear much more frequently than official data reflect. Furthermore, the results of our study showed, to our knowledge, for the first time, CAEV infection in sheep, detected by an immunohistochemical method. During the last 10 years, MVV was reported in single cases. Nevertheless, a mild increase in MVV cases was also seen from mid-2017 to the end of 2019.

The JSRV-positive lungs, detected by IHC or/and PCR, showed typical pathomorphological lesions, as described in the literature [1,28,33,39]. In addition to detection of viral antigen in proliferating bronchiolar epithelial cells, the viral antigen was shown in the cytoplasm of macrophages. In contrast to Griffiths et al. [25], viral antigen was not only detected in neoplastic epithelial cells but also in macrophages, which could be explained by the phagocytic activity of those cells. In an ultrastructural study, ingested JSRV particles in the cytoplasm of macrophages [40] were described, which may explain, partially, the immunoreactivity of those cells in our study. Nevertheless, macrophages as possible sites of viral replication cannot be ruled out. Like the study of Martineau et al. [1], only a small number of cells were positive for SP-C but negative for synaptophysin, thus, demonstrating that alveolar type II pneumocytes are a target cell population in JSRV infection.

In our ovine lung tissues, morphological lesions typical for SRLV infections were observed in 25% of all the 97 examined cases, while only 24% of these samples were positive for MVV and/or CAEV by PCR. On the other hand, SRLV infection could be detected by PCR in 10% of the lungs that were classified as not having histological lesions compatible with an SRLV infection. These results suggest a higher sensitivity of the PCR assay compared to the histological examination alone to detect SRLV infections.

In MVV IHC, viral antigen was localized mainly in the cytoplasm of macrophages, confirming the results of a previous study [41]. In our study, CAEV antigen was most frequently identified in bronchial epithelial cells with a strong cytoplasmatic and nuclear signal in areas of lymphocytic infiltration, in contrast to the findings of Storset et al. [42], who detected the signal mainly in the cytoplasm of macrophages. The immunoreactivity of bronchial epithelial cells is an uncommon finding, which was not described by other authors. Nevertheless, the infection of different epithelial subtypes, such as mammary epithelial cells or uterine epithelial cells, is known [43,44].

Some cases which showed histological lesions compatible with SRLV infection displayed a negative PCR result. Nevertheless, a small percentage of macrophage/epithelial cells revealed immunoreactivity for MVV or CAEV, respectively. Most of these samples were archival lungs and the PCR was conducted on paraffin-embedded material, which eventually, due to long preservation time, led to negative results. These results indicate that the time of formalin fixation and paraffin embedding could result in a negative PCR outcome.

All the commercially available antibodies that detect SRLVs do not differentiate between MVV and CAEV due to the close relationship of these viruses. Peptide antibodies represent a relatively new tool for detection of specific antigenic structures [45]. This new technique was, for example, used in Dengue fever patients’ sera [46].

Co-infections with different viruses were of low prevalence but did occur [32], making it more difficult to diagnose them by only one method. Therefore, the use of a combined approach to diagnose these notifiable diseases is recommended. In a recent paper [32], the combination of ELISA and PCR in serological samples showed co-infection of sheep and goats with SRLV. A co-infection with SRLVs in goats was also described by Pisoni et al. [47]. Five main genotypes (A–E) and more than 28 subgroups of SRLVs have already been characterized [30]. Genotype A in sheep consists of MVV-like strains, whereas genotype B comprises CAEV-like isolates [48]. Due to the proposed genetic continuum, cross-species infection may occur [49]. This explains the occurrence of CAEV infected sheep in our study. Due to the low caprine case load at our institute, a detailed investigation of SRLV in goats was not applicable. In further studies, a survey, including a large number of caprine samples, should be implemented. 

In two cases, neoplastic epithelial cells could be detected in regional lymph nodes in OPA cases. If the slaughter process reveals evidence of metastases, the carcass is not released for human consumption. In such cases, the human consumption of the meat must be reconsidered and the meat confiscated. The metastatic potential of OPA should not be underestimated.

## 5. Conclusions

In this study, the presence of JSRV, CAEV and MVV as single infections as well as co-infections in sheep in Switzerland was confirmed by PCR and IHC. The newly developed and established immunohistochemical protocols provide a useful tool for the specific and differential detection of retroviral antigen in routinely formalin fixed and paraffin-embedded ovine tissue. Although PCR has a higher sensitivity than IHC, the combined approach allows for investigations on viral cell tropism and pathogenic processes in co-morbidities, including their potential interdependency.

## Figures and Tables

**Table 1:** Antibody used in immunohistochemistry.

Antibody	Company/Item Number	Dilution	Pretreatment
PCK 26 ^1^	Novus Biologicals, USA/NB120-6401	1:10,000	Pressure cooker pH9
Synaptophysin	Agilent Dako, USA/M077601	1:50	Pressure cooker pH9
Iba1	FUJIFILM Wako Pure Chemical Corporation, USA/ 019-19741	1:1000	Pressure cooker pH6
SF-C ^2^	Abcam, UK/Ab40879	1:4000	Pressure cooker pH9
CD3	Agilent Dako, USA/M725401	1:50	Pressure cooker pH9
CD20	Thermo Fisher Scientific, USA/RB-9013-P	1:100	Pressure cooker pH9
Polyclonal rabbit anti-JSRV ^3^	Thermo Fisher Scientific, USA/AB3994 (customized antibody)	1:500	none
Polyclonal rabbit anti-MVV ^4^	Thermo Fisher Scientific, USA/AB 3989 (customized antibody)	1:100	Pressure cooker pH6
Polyclonal rabbit anti-CAEV ^5^	Thermo Fisher Scientific, USA/AB3992 (customized antibody)	1:200	Pressure cooker pH6

^1^ pan-cytokeratin, ^2^ surfactant protein C, ^3^ Jaagsiekte sheep retrovirus, ^4^ maedi-visna virus, ^5^ caprine arthritis encephalitis virus, customized by Thermo Fisher Scientific.

**Table 2:** Summary of the results of the nested real-time PCR.

Detection of	Number of Animals (N = 97)
JSRV only	37
MVV only	5
CAEV only	0
JSRV/MVV	6
JSRV/CAEV	1
MVV/CAEV	3
negative	45

**Table 3:** Cell markers used in IHC and their relevance in small ruminant retrovirus cases.

Marker	Cell Population	Small Ruminant Retrovirus Cases ^1^
PCK 26	Epithelial cells	Infected cell population in JSRV (+++), MVV (+) and CAEV (++); lymph node metastases in OPA
Synaptophysin	Neuroendocrine cells	Not infected
Iba1	Macrophages	Infected cell population in JSRV (++), MVV (++) and CAEV (+)
SP-C	Type II pneumocytes	Infected cell population in JSRV (+), MVV (+), CAEV (+)

^1^ + = small number; ++ = moderate number; +++ = large number.

## Data Availability

Not applicable.

## Supplementary Materials

The following supporting information can be downloaded at: https://www.mdpi.com/article/10.3390/v15020376/s1, Figure S1: Data from the BLV (Bundesamt für Lebensmittelsicherheit und Veterinärwesen; Federal Food Safety and Veterinary Office, Switzerland); Figure S2 [50]: Se-quence-based peptide analysis for (a) CAEV, (b) MVV, and (c) exJSRV; Figure S3: Double im-munohistology to identify viral antigens and cell marker; Figure S4: Negative controls in immunohistochemistry.

## References

[1] Martineau H.M., Cousens C., Imlach S., Dagleish M.P., Griffiths D.J. (2011). Jaagsiekte Sheep Retrovirus Infects Multiple Cell Types in the Ovine Lung. J. Virol..

[2] Gayo E., Polledo L., Balseiro A., Martínez C.P., García Iglesias M.J., Preziuso S., Rossi G., García Marín J.F. (2018). Inflammatory Lesion Patterns in Target Organs of Visna/Maedi in Sheep and Their Significance in the Pathogenesis and Diagnosis of the Infection. J. Comp. Pathol..

[3] Leroux C., Chastang J., Greenland T., Mornex J.F. (1997). Genomic Heterogeneity of Small Ruminant Lentiviruses: Existence of Heterogeneous Populations in Sheep and of the Same Lentiviral Genotypes in Sheep and Goats. Arch. Virol..

[4] Germain K., Valas S. (2006). Distribution and Heterogeneity of Small Ruminant Lentivirus Envelope Subtypes in Naturally Infected French Sheep. Virus Res..

[5] Pisoni G., Bertoni G., Boettcher P., Ponti W., Moroni P. (2006). Phylogenetic Analysis of the Gag Region Encoding the Matrix Protein of Small Ruminant Lentiviruses: Comparative Analysis and Molecular Epidemiological Applications. Virus Res..

[6] Da Cruz J.C.M., Singh D.K., Lamara A., Chebloune Y. (2013). Small Ruminant Lentiviruses (SRLVs) Break the Species Barrier to Acquire New Host Range. Viruses.

[7] Anderson L.W., Klevjer-Anderson P., Liggitif H.D. (1983). Susceptibility of Blood-Derived Monocytes and Macrophages to Caprine Arthritis-Encephalitis Virus. Infect. Immun..

[8] Narayan O., Kennedy-Stoskopf S., Sheffer D., Griffin D.E., Clements J.E. (1983). Activation of Caprine Arthritis-Encephalitis Virus Expression During Maturation of Monocytes to Macrophages. Infect. Immun..

[9] Narayan O., Wolinsky J.S., Clements J.E., Strandberg J.D., Griffin D.E., Cork L.C. (1982). Slow Virus Replication: The Role of Macrophages in the Persistence and Expression of Visna Viruses of Sheep and Goats. J. Gen. Virol..

[10] Gendelman H.E., Narayan O., Kennedy-Stoskopf S., Kennedy P.G.E., Ghotbi Z., Clements J.E., Stanley J., Pezeshkpour G. (1986). Tropism of Sheep Lentiviruses for Monocytes: Susceptibility to Infection and Virus Gene Expression Increase during Maturation of Monocytes to Macrophages. J. Virol..

[11] Ryan S., Tiley L., Mcconnell I., Blacklaws B. (2000). Infection of Dendritic Cells by the Maedi-Visna Lentivirus. J. Virol..

[12] Zink M.C., Yager J.A., Myers J.D. (1990). Pathogenesis of Caprine Arthritis Encephalitis Virus Cellular Localization of Viral Transcripts in Tissues of Infected Goats. Am. J. Pathol..

[13] Lerondelle C., Godet M., Mornex J.F. (1999). Infection of Primary Cultures of Mammary Epithelial Cells by Small Ruminant Lentiviruses. Vet. Res..

[14] Blacklaws B.A., Berriatua E., Torsteinsdottir S., Watt N.J., de Andres D., Klein D., Harkiss G.D. (2004). Transmission of Small Ruminant Lentiviruses. Vet. Microbiol..

[15] McNeilly T.N., Tennant P., Luján L., Pérez M., Harkiss G.D. (2007). Differential Infection Efficiencies of Peripheral Lung and Tracheal Tissues in Sheep Infected with Visnal/Maedi Virus via the Respiratory Tract. J. Gen. Virol..

[16] Niesalla H., McNeilly T.N., Ross M., Rhind S.M., Harkiss G.D. (2008). Experimental Infection of Sheep with Visna/Maedi Virus via the Conjunctival Space. J. Gen. Virol..

[17] McNeilly T.N., Baker A., Brown J.K., Collie D., MacLachlan G., Rhind S.M., Harkiss G.D. (2008). Role of Alveolar Macrophages in Respiratory Transmission of Visna/Maedi Virus. J. Virol..

[18] Peterhans E., Greenland T., Badiola J., Harkiss G., Bertoni G., Amorena B., Eliaszewicz M., Juste R.A., Kraßnig R., Lafont J.P. (2004). Routes of Transmission and Consequences of Small Ruminant Lentiviruses (SRLVs) Infection and Eradication Schemes. Vet. Res..

[19] Benavides J., García-Pariente C., Fuertes M., Ferreras M.C., García-Marín J.F., Juste R.A., Pérez V. (2009). Maedi-Visna: The Meningoencephalitis in Naturally Occurring Cases. J. Comp. Pathol..

[20] Minguijón E., Reina R., Pérez M., Polledo L., Villoria M., Ramírez H., Leginagoikoa I., Badiola J.J., García-Marín J.F., de Andrés D. (2015). Small Ruminant Lentivirus Infections and Diseases. Vet. Microbiol..

[21] Jeff L., Caswell K.J.W. (2007). Respiratory System. Jubb, Kennedy & Palmer’s Pathology of Domestic Animals.

[22] Salvatori D., González L., Dewar P., Cousens C., de las Heras M., Dalziel R.G., Sharp J.M. (2004). Successful Induction of Ovine Pulmonary Adenocarcinoma in Lambs of Different Ages and Detection of Viraemia during the Preclinical Period. J. Gen. Virol..

[23] Caporale M., Centorame P., Giovannini A., Sacchini F., di Ventura M., de Las Heras M., Palmarini M. (2005). Infection of Lung Epithelial Cells and Induction of Pulmonary Adenocarcinoma Is Not the Most Common Outcome of Naturally Occurring JSRV Infection during the Commercial Lifespan of Sheep. Virology.

[24] Grego E., de Meneghi D., Álvarez V., Benito A.A., Minguijón E., Ortín A., Mattoni M., Moreno B., Pérez de Villarreal M., Alberti A. (2008). Colostrum and Milk Can Transmit Jaagsiekte Retrovirus to Lambs. Vet. Microbiol..

[25] Griffiths D.J., Martineau H.M., Cousens C. (2010). Pathology and Pathogenesis of Ovine Pulmonary Adenocarcinoma. J. Comp. Pathol..

[26] Holland M.J., Palmarini M., Garcia-Goti M., Gonzalez L., McKendrick I., de las Heras M., Sharp J.M. (1999). Jaagsiekte Retrovirus Is Widely Distributed Both in T and B Lymphocytes and in Mononuclear Phagocytes of Sheep with Naturally and Experimentally Acquired Pulmonary Adenomatosis. J. Virol..

[27] Scott P., Griffiths D., Cousens C. (2013). Diagnosis and Control of Ovine Pulmonary Adenocarcinoma (Jaagsiekte). Practice.

[28] Mishra S., Kumar P., Dar J.A., George N., Singh V., Singh R. (2021). Differential Immunohistochemical Expression of JSRV Capsid Antigen and Tumour Biomarkers in Classical and Atypical OPA: A Comparative Study. Biol. Rhythm Res..

[29] Michiels R., van Mael E., Quinet C., Adjadj N.R., Cay A.B., de Regge N. (2018). Comparative Analysis of Different Serological and Molecular Tests for the Detection of Small Ruminant Lentiviruses (Srlvs) in Belgian Sheep and Goats. Viruses.

[30] Michiels R., Adjadj N.R., de Regge N. (2020). Phylogenetic Analysis of Belgian Small Ruminant Lentiviruses Supports Cross Species Virus Transmission and Identifies New Subtype B5 Strains. Pathogens.

[31] Thomann B., Falzon L.C., Bertoni G., Vogt H.R., Schüpbach-Regula G., Magouras I. (2017). A Census to Determine the Prevalence and Risk Factors for Caprine Arthritis-Encephalitis Virus and Visna/Maedi Virus in the Swiss Goat Population. Prev. Vet. Med..

[32] Schaer J., Cvetnic Z., Sukalic T., Dörig S., Grisiger M., Iscaro C., Feliziani F., Pfeifer F., Origgi F., Zanoni R.G. (2022). Evaluation of Serological Methods and a New Real-Time Nested PCR for Small Ruminant Lentiviruses. Pathogens.

[33] Palmarini M., Holland M.J., Cousens C., Dalziel R.G., Sharp J.M. (1996). Jaagsiekte Retrovirus Establishes a Disseminated Infection of the Lymphoid Tissues of Sheep Affected by Pulmonary Adenomatosis. J. Gen. Virol..

[34] Liu S.-L., Miller A.D. (2007). Oncogenic Transformation by the Jaagsiekte Sheep Retrovirus Envelope Protein. Oncogene.

[35] Zhang K., Kong H., Liu Y., Shang Y., Wu B., Liu X. (2014). Diagnosis and Phylogenetic Analysis of Ovine Pulmonary Adenocarcinoma in China. Virus Genes.

[36] Bahari A., Ghannad M.S., Dezfoulian O., Rezazadeh F., Sadeghi-Nasab A. (2016). Detection of Jaagsiekte Sheep Retrovirus in Apparently Healthy Sheep by Real-Time TaqMan PCR in Comparison with Histopathological Findings. J. Vet. Res..

[37] Liu Y., Zhang Y.F., Sun X.L., Liu S.Y. (2016). Detection of Jaagsiekte Sheep Retrovirus in the Peripheral Blood during the Pre-Clinical Period of Ovine Pulmonary Adenomatosis. Genet. Mol. Res..

[38] Lee A.M., Wolfe A., Cassidy J.P., Messam L.M.V.L., Moriarty J.P., O’Neill R., Fahy C., Connaghan E., Cousens C., Dagleish M.P. (2017). First Confirmation by PCR of Jaagsiekte Sheep Retrovirus in Ireland and Prevalence of Ovine Pulmonary Adenocarcinoma in Adult Sheep at Slaughter. Ir. Vet. J..

[39] DeMartini J.C., Bishop J.V., Allen T.E., Jassim F.A., Sharp J.M., de las Heras M., Voelker D.R., Carlson J.O. (2001). Jaagsiekte Sheep Retrovirus Proviral Clone JSRV(JS7), Derived from the JS7 Lung Tumor Cell Line, Induces Ovine Pulmonary Carcinoma and Is Integrated into the Surfactant Protein A Gene. J. Virol..

[40] Payne A.L., Verwoerd D.W. (1984). A Scanning and Transmission Electron Microscopy Study of Jaagsiekte Lesions. Onderstepoort. J. Vet. Res..

[41] Kumar K., Kumar P., Sindhoora K., Valecha S., Kumar R., Singh V., Singh R. (2022). Detection and Immune Cell Response of Natural Maedi Visna Virus (MVV) Infection in Indian Sheep and Goats. Microb. Pathog..

[42] Storset A.K., Evensen Ø., Rimstad E. (1997). Immunohistochemical Identification of Caprine Arthritis-Encephalitis Virus in Paraffin-Embedded Specimens from Naturally Infected Goats. Vet. Pathol..

[43] Blacklaws B.A. (2012). Small Ruminant Lentiviruses: Immunopathogenesis of Visna-Maedi and Caprine Arthritis and Encephalitis Virus. Comp. Immunol. Microbiol. Infect. Dis..

[44] Ali Al Ahmad M.Z., Dubreil L., Chatagnon G., Khayli Z., Theret M., Martignat L., Chebloune Y., Fieni F. (2012). Goat Uterine Epithelial Cells Are Susceptible to Infection with Caprine Arthritis Encephalitis Virus (CAEV) in Vivo. Vet. Res..

[45] Trier N., Hansen P., Houen G. (2019). Peptides, Antibodies, Peptide Antibodies and More. Int. J. Mol. Sci..

[46] Bergamaschi G., Fassi E.M.A., Romanato A., D’Annessa I., Odinolfi M.T., Brambilla D., Damin F., Chiari M., Gori A., Colombo G. (2019). Computational Analysis of Dengue Virus Envelope Protein (E) Reveals an Epitope with Flavivirus Immunodiagnostic Potential in Peptide Microarrays. Int. J. Mol. Sci..

[47] Pisoni G., Bertoni G., Puricelli M., Maccalli M., Moroni P. (2007). Demonstration of Coinfection with and Recombination by Caprine Arthritis-Encephalitis Virus and Maedi-Visna Virus in Naturally Infected Goats. J. Virol..

[48] Ramírez H., Reina R., Amorena B., Andrés D., Martínez H. (2013). Small Ruminant Lentiviruses: Genetic Variability, Tropism and Diagnosis. Viruses.

[49] Leroux C., Cruz J.C.M., Mornex J.-F. (2010). SRLVs: A Genetic Continuum of Lentiviral Species in Sheep and Goats with Cumulative Evidence of Cross Species Transmission. Curr. HIV Res..

[50] Dhanda S.K., Vaughan K., Schulten V., Grifoni A., Weiskopf D., Sidney J., Peters B., Sette A. (2018). Development of a Novel Clustering Tool for Linear Peptide Sequences. Immunology.

