# Supplementary material for: A Combined Approach for Detection of Ovine Small Ruminant Retrovirus Co-Infections
Source: Viruses. 2023 Jan 28;15(2):376. doi: 10.3390/v15020376 (PMC9958757; doi:10.3390/v15020376)
Supplement: Supplementary file 1 [file viruses-15-00376-s001.zip › Supplementary Figure S1.pdf]

a)

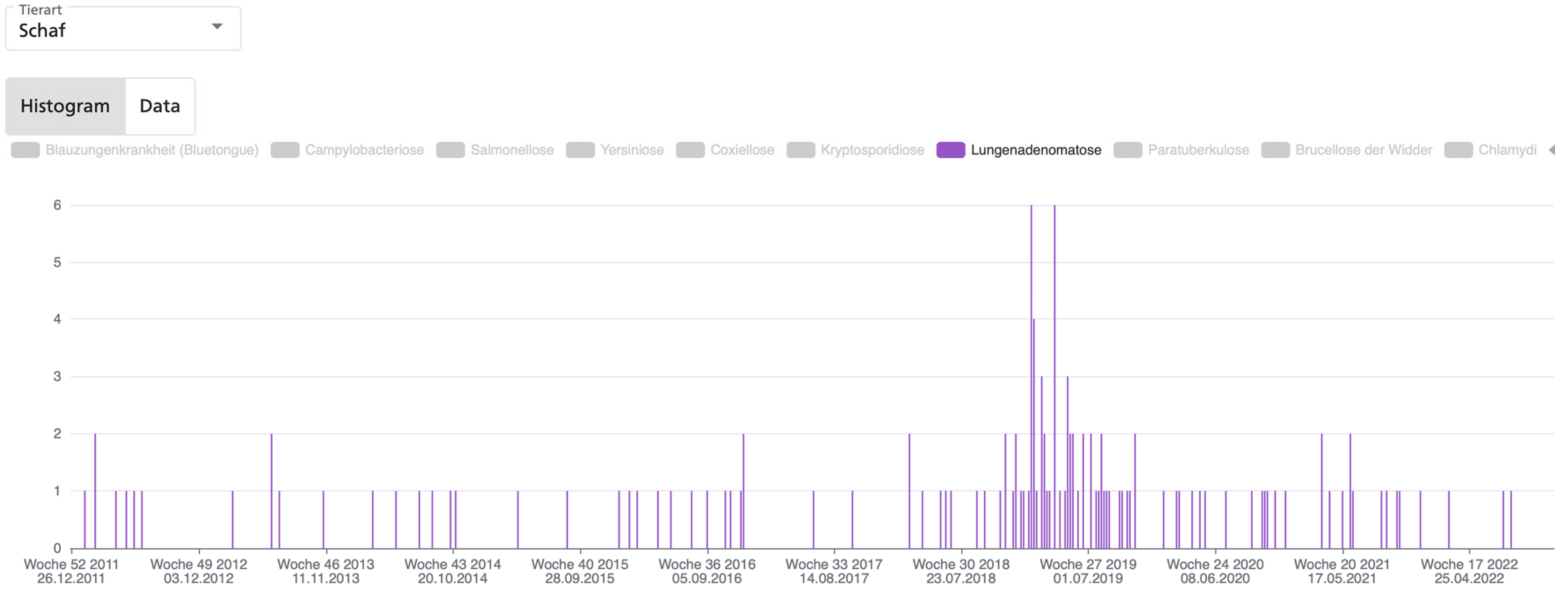

Tierart
Schaf
Histogram
Data
Blauzungenkrankheit (Bluetongue)
Campylobacteriose
Salmonellose
Yersiniose
Coxiellose
Kryptosporidiose
Lungenadenomatose
Paratuberkulose
Brucellose der Widder
Chlamydi
6
5
4
3
2
1
0
Woche 52 2011 26.12.2011
Woche 49 2012 03.12.2012
Woche 46 2013 11.11.2013
Woche 43 2014 20.10.2014
Woche 40 2015 28.09.2015
Woche 36 2016 05.09.2016
Woche 33 2017 14.08.2017
Woche 30 2018 23.07.2018
Woche 27 2019 01.07.2019
Woche 24 2020 08.06.2020
Woche 20 2021 17.05.2021
Woche 17 2022 25.04.2022

b)

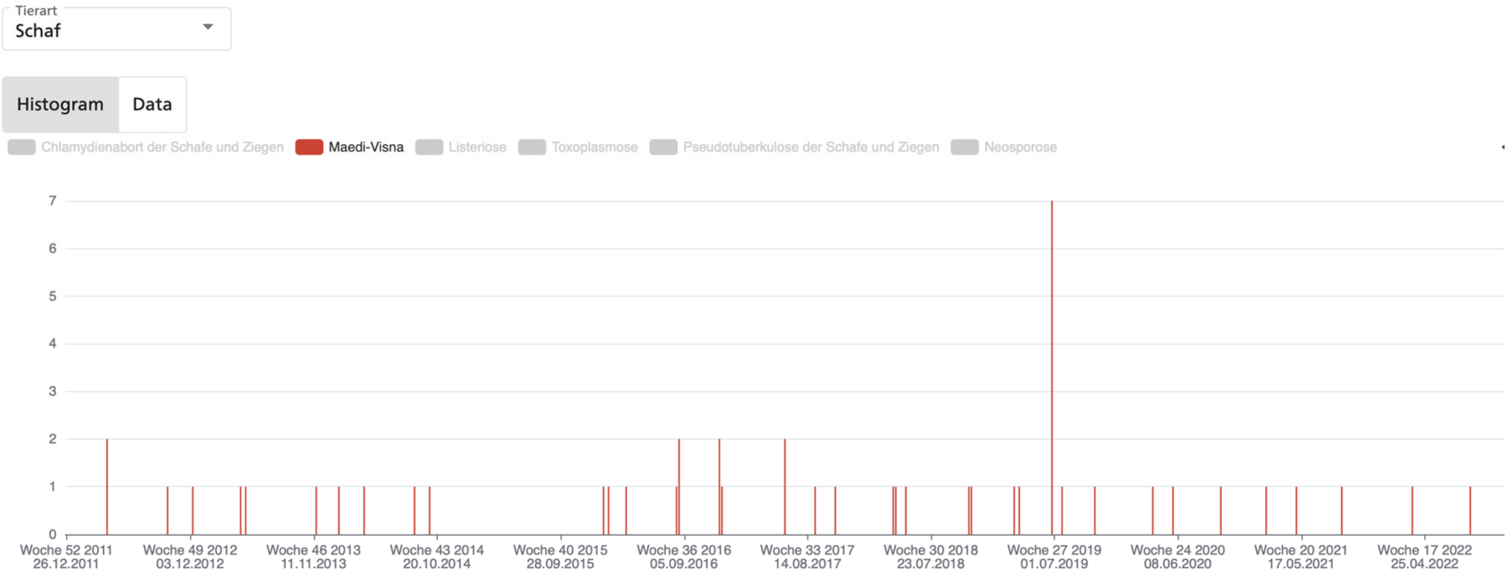

Supplementary Figure S1: Data from the BLV (Bundesamt für Lebensmittelsicherheit und Veterinärwesen; Federal Food Safety and Veterinary Office, Switzerland). Figures show ovine OPA cases (a) and maedi visna cases (b) from 2012 to 2022. During the period of investigation of slaughterhouse samples an increase of positive cases was noted (especially in 2019).

https://www.infosm.blv.admin.ch/evaluation/animal
