# Supplementary material for: A Combined Approach for Detection of Ovine Small Ruminant Retrovirus Co-Infections
Source: Viruses. 2023 Jan 28;15(2):376. doi: 10.3390/v15020376 (PMC9958757; doi:10.3390/v15020376)
Supplement: Supplementary file 1 [file viruses-15-00376-s001.zip › Supplementary Figure S2.pdf]

(a) CAEV peptide sequence analysis.

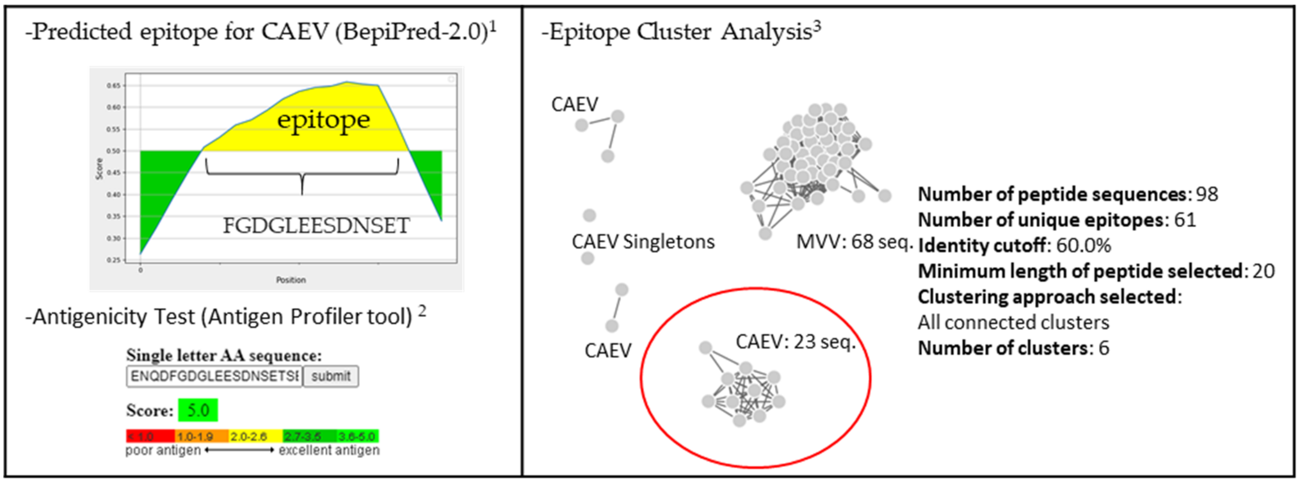

(b) MVV peptide sequence analysis.

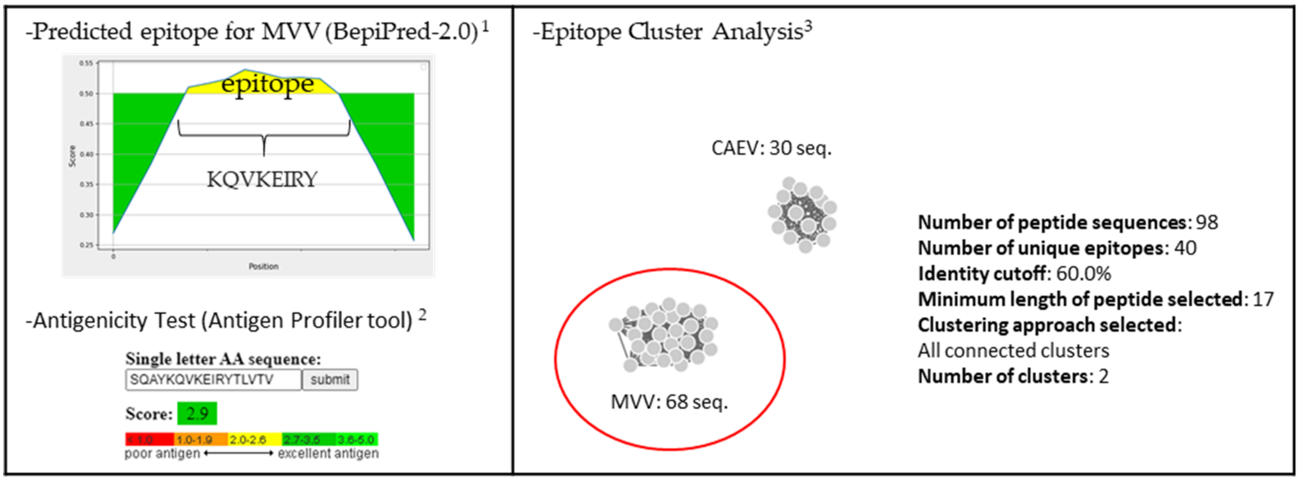

(c) exJSRV peptide sequence analysis.

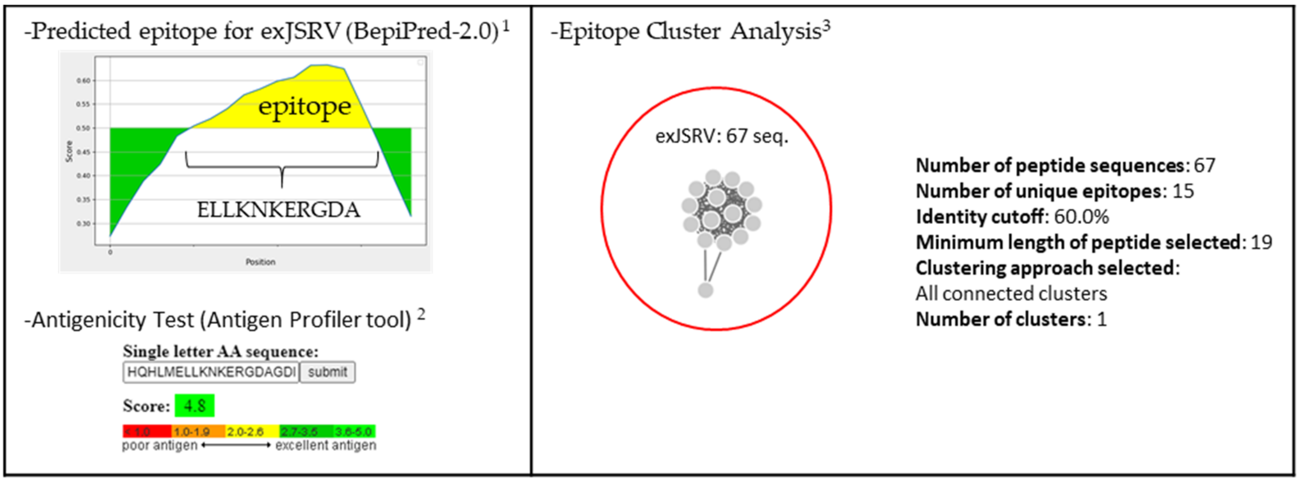

Supplementary Figure S2. Sequence-based peptide analysis for (a) CAEV, (b) MVV, and (c) exJSRV. [1] Conserved protein sequence regions were analyzed for the identification of antigenic peptides (BepiPred-2.0 prediction tool, http://www.iedb.org/home_v3.php) Dhanda et al. [2] The Antigen Profiler Peptide Tool (https://www.thermofisher.com/ch/en/home/life-science/antibodies/custom-antibodies/custom-antibody-production/antigen-profiler-antigen-preparation.html) was additionally used for confirmation of the antigenicity.

[3] Epitope cluster analysis (http://www.iedb.org/home_v3.php) based on sequence identity was used to estimate and visualized the conservancy of the epitope regions. These conservancy analyses included the envelope protein sequences of 30, 68 and 67 CAEV, MVV and the exJSRV, respectively. Peptides are presented as circles and line connecting two circles means the two peptides shared identity above given threshold. Clumped cluster means high interconnectivity among the peptides. The localization in the clusters of the selected peptides for the production of antibodies is indicated with red circles.

Reference

50. Dhanda, S.K.; Vaughan, K.; Schulten, V.; Grifoni, A.; Weiskopf, D.; Sidney, J.; Peters, B.; Sette, A. Development of a novel clustering tool for linear peptide sequences. *Immunology* **2018**, 155(3), 331-345, doi: 10.1111/imm.12984.
