# Supplementary material for: A Combined Approach for Detection of Ovine Small Ruminant Retrovirus Co-Infections
Source: Viruses. 2023 Jan 28;15(2):376. doi: 10.3390/v15020376 (PMC9958757; doi:10.3390/v15020376)
Supplement: Supplementary file 1 [file viruses-15-00376-s001.zip › Supplementary Figure S3.pdf]

a)

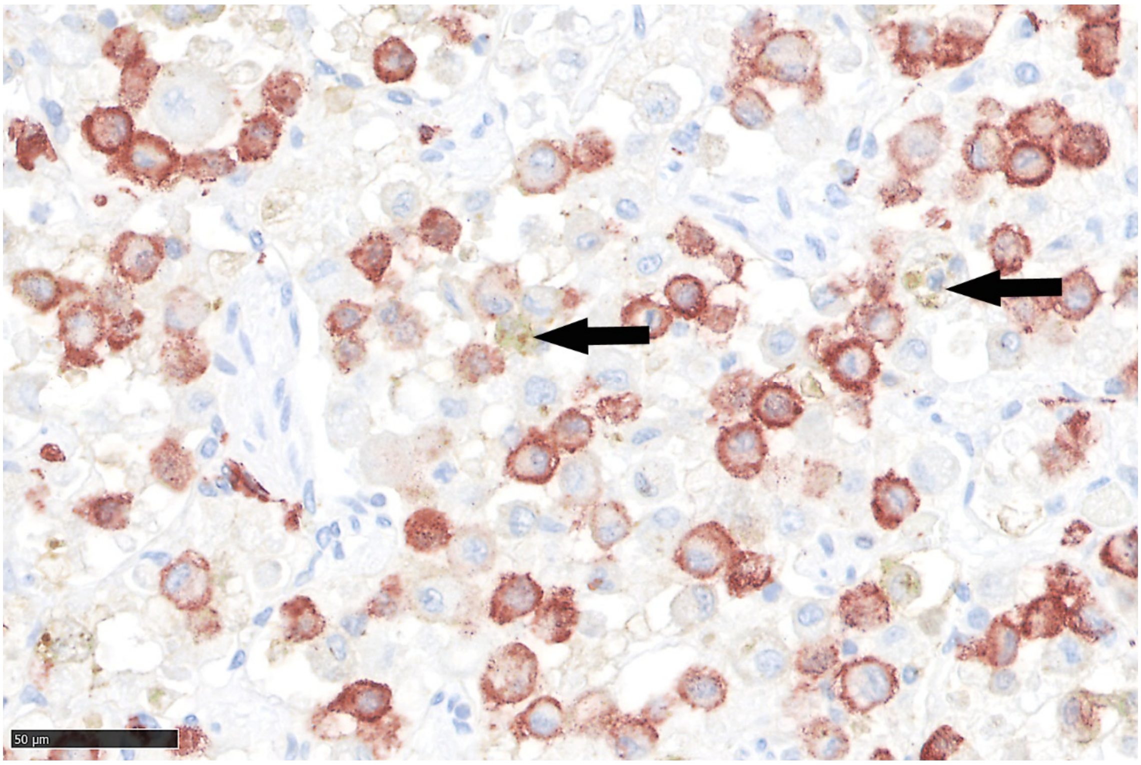

b)

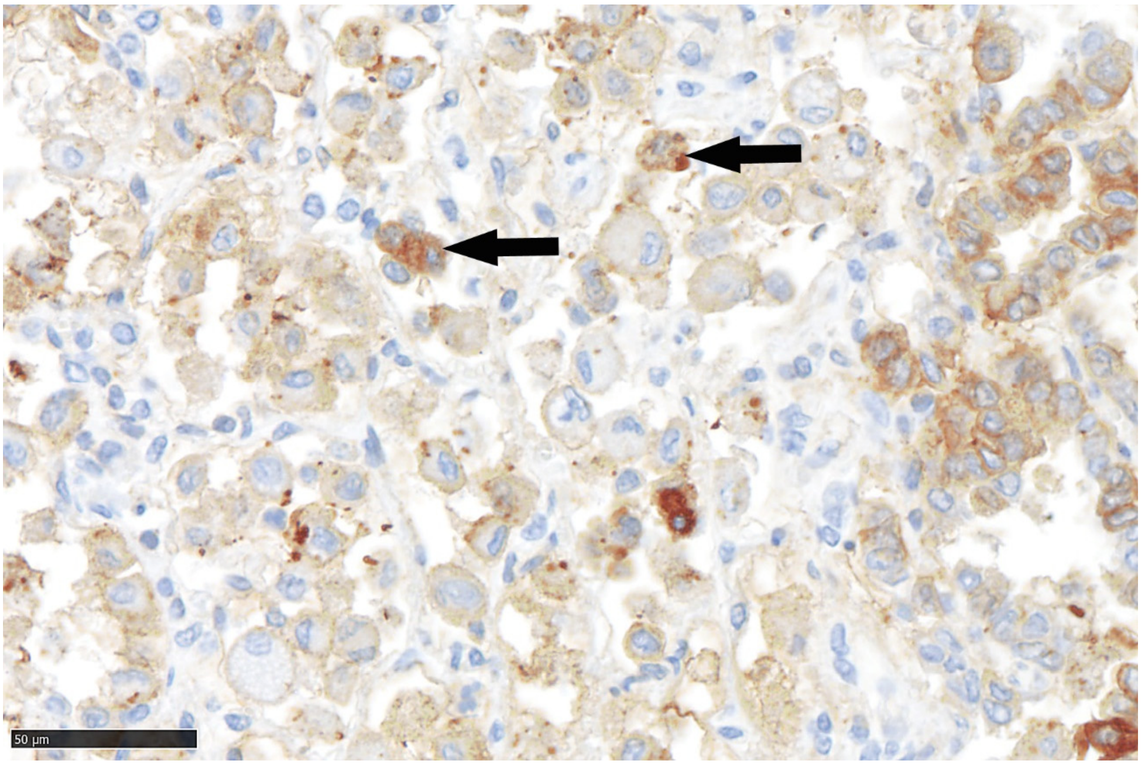

c)

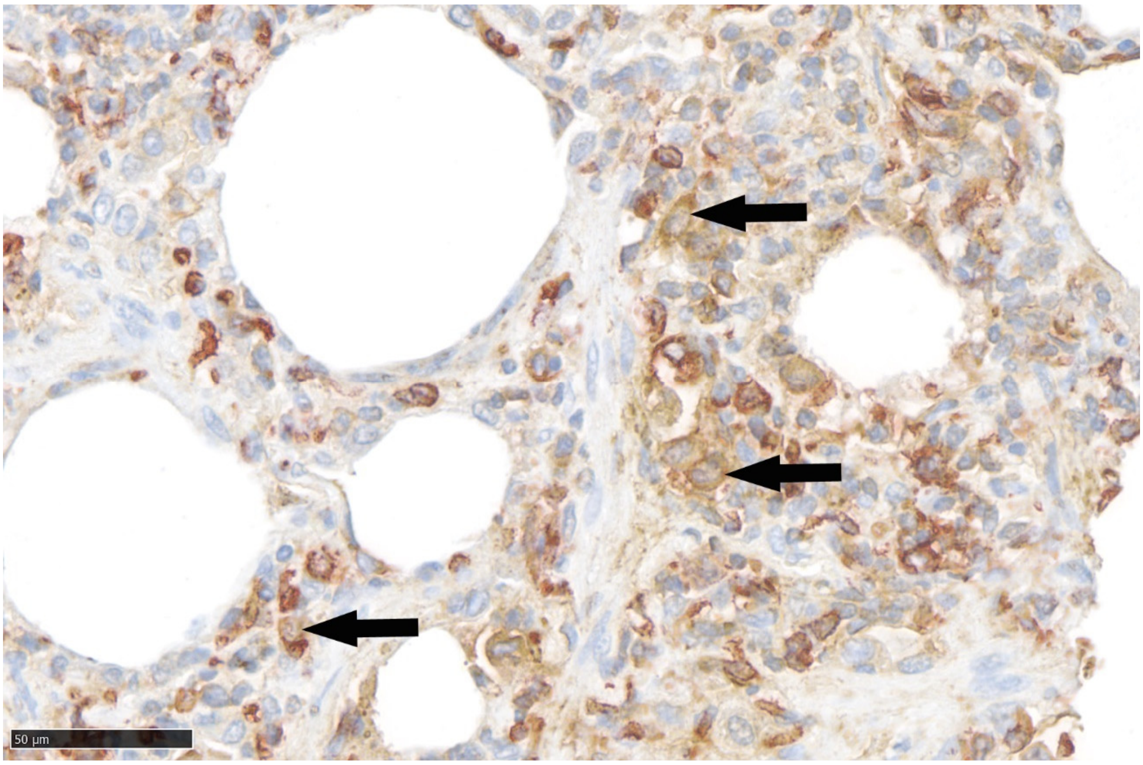

Supplementary Figure S3: Double immunohistology to identify viral antigens and cell marker. a) JSRV (green)/Iba1 (red) double IHC depicting a moderate number of Iba1 positive cells with a histiocytic morphology and colocalization of viral antigen which results in a brown-reddish mixed color. b) JSRV (green)/SP-C (red) double IHC depicting a small number of SP-C positive type II pneumocytes with colocalization of viral antigen which results in a brown-reddish mixed color. c) MVV (green)/Iba1 (red) double IHC depicting a moderate number of Iba1 positive cells with a histiocytic morphology and colocalization of viral antigen which results in a brown-reddish mixed color.
