# Supplementary material for: A Combined Approach for Detection of Ovine Small Ruminant Retrovirus Co-Infections
Source: Viruses. 2023 Jan 28;15(2):376. doi: 10.3390/v15020376 (PMC9958757; doi:10.3390/v15020376)
Supplement: Supplementary file 1 [file viruses-15-00376-s001.zip › Supplementary Figure S4.pdf]

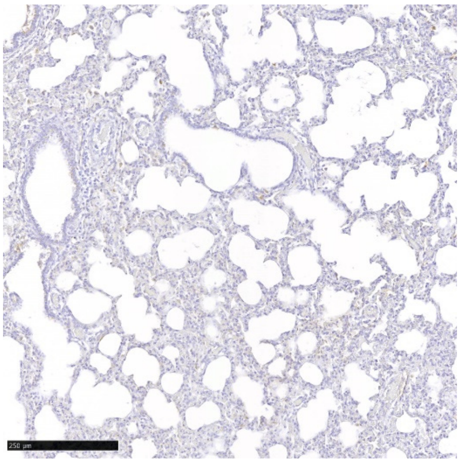

(**a**)

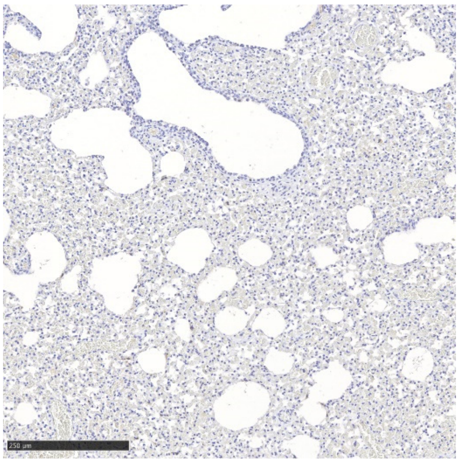

(**b**)

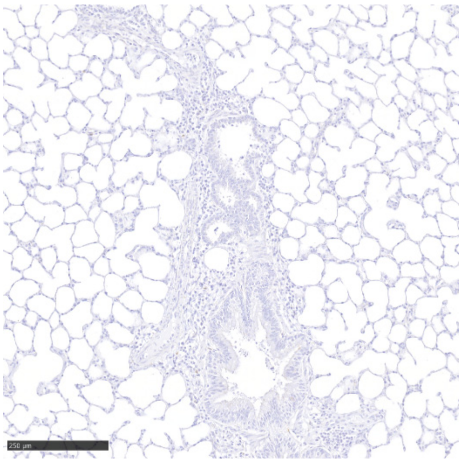

(c)

Supplementary Figure S4. Negative controls in immunohistochemistry (a) No immunoreactivitiy for JSRV antigen in pulmonary tissue of an animal without lesions. (b) No immunoreactivitiy for MVV antigen in an ovine lung with a purulent pneumonia (c) No immunoreactivitiy for CAEV antigen in a lung without pulmonary lesions. Chromogen DAB. Bar = 250 μm. All animals were PCR negative.
